# l-Lactate Transport and Metabolism in Mitochondria of Hep G2 Cells—The Cori Cycle Revisited

**DOI:** 10.3389/fonc.2018.00120

**Published:** 2018-04-23

**Authors:** Salvatore Passarella, Avital Schurr

**Affiliations:** ^1^School of Medicine University “Aldo Moro”, Piazza Giulio Cesare, Bari, Italy; ^2^Department of Anesthesiology and Perioperative Medicine, University of Louisville School of Medicine, Louisville, KY, United States

**Keywords:** l-lactate, l-lactate dehydrogenase, mitochondria, cancer, Cori cycle, mitochondrial transport

In addition to being a glucose precursor in liver and kidney, l-lactate is now also being recognized as an energy substrate in most cells *via* its oxidation to pyruvate. This oxidation, assumed to occur in the cytosol, is catalyzed by l-lactate dehydrogenase with pyruvate subsequently catabolized in the mitochondria. However, recently mitochondria were recognized to play a role in l-lactate metabolism: the existence of a mitochondrial l-lactate dehydrogenase (m-l-LDH) was suggested by Dianzani (1), and later demonstrated by Baba and Sharma (2) to be located in the mitochondrial matrix (3). Indeed, l-lactate transport and metabolism was shown in various mitochondria, including skeletal muscle (4) rat heart (5), liver (6), brain (7–9), cerebellar granule cells (10), rabbit gastrocnemius (11), sperm cells (12), pig liver (13), and even plant (14). Thus, the existence of m-l-LDH, as reviewed by Passarella et al. (3), Brooks (15), and Schurr (16), was recognized with its inclusion in the MitoCarta (http://www.broadinstitute.org/pubs/MitoCarta/index.htrnl). As expected, in light of the presence of the l-LDH in the matrix, the occurrence of carriers for l-lactate has been shown in functional studies with purified, coupled mitochondria. These include the l-lactate/H^+^ symporter and the l-lactate/pyruvate and l-lactate/oxaloacetate antiporters (3). Surprisingly, the overwhelming evidence for an m-l-LDH located inside mitochondria is not universally accepted, with some scientists still being skeptic about the existence of m-l-LDH, while others localizing m-l-LDH in the intermembrane space (17). It is our opinion that the skepticism could originate due to difficulties in isolating coupled mitochondria, not an easy task, in particular with skeletal muscle samples, or not being careful enough in selecting reaction media and in using inhibitors at the correct concentration (11). That m-l-LDH is localized inside mitochondria will be shown below.

## Is l-Lactate Being Transported and Metabolized in Cancer Cell Mitochondria?

Yes, it is. Although in the 1920s, Warburg found that cancer cells prefer to produce ATP by glycolysis with l-lactate production, to the best of our knowledge, the mitochondrial metabolism of l-lactate had not been investigated in cancer cells until 2010, when the first evidence for l-lactate mitochondrial metabolism in these cells (already reported in 2008 by Gabriella Chieppa in her PhD thesis at the University of Molise) was published (18). In this case, to study l-lactate transport and metabolism in mitochondria isolated from both normal and cancer prostate cells, spectroscopic and polarographic techniques were used, in which either m-l-LDH reaction or oxygen consumption by mitochondria, supplied with externally added l-lactate were monitored, respectively (19), rather than employing more involved procedures, available in molecular biology, genetics, and chemistry laboratories. The former two techniques were chosen since they afford the continuous monitoring of the kinetics of the investigated processes in experiments that last for several minutes where mitochondria remain coupled. By contrast, measurements using the latter methods are usually made once the processes have already been completed. Accordingly, an increase in the redox state of the intramitochondrial pyridine nucleotides, as shown by fluorimetric measurements, upon the addition of l-lactate to mitochondria indicates that l-lactate metabolism occurs inside the organelles *via* an NAD^+^-dependent m-l-LDH; unfortunately, the occurrence of the mitochondrial l-lactate metabolism in cancer cells was not quoted in Ferguson et al. (17) possibly because the authors of the review consider the spectroscopic and polarographic techniques to be “problematic,” despite its widespread use by numerous scientists. That theirs is a minority opinion might be exemplified by quoting from a review by Mayevsky and Rogatsky (20), which states that “The large numbers of publications by different groups testify to the valuable information gathered in various experimental conditions. The monitoring of NADH levels in the tissue provides the most important information on the metabolic state of the mitochondria.” The existence of m-l-LDH can be also immunologically confirmed in mitochondria that are proven to be free of cytosolic contamination.

Notice that in the case where m-l-LDH is proposed to be localized in the intermembrane space, the increase in the intramitochondrial pyridine nucleotide fluorescence is explained as follows: l-lactate enters the mitochondrial intermembrane space where it is oxidized to pyruvate, which in turn crosses the mitochondrial inner membrane to be oxidized inside the mitochondria *via* the pyruvate dehydrogenase complex [for review, see Ferguson et al. (17)]. Such a mechanism is not supported by various experimental findings. For instance, in de Bari et al. (18), it was shown that NAD^+^ reduction proceeds despite the presence of arsenite, an inhibitor of pyruvate dehydrogenase, but is inhibited by oxamate, an inhibitor of l-LDH. Additional evidence against the presence of m-l-LDH in the intermembrane space emerges from experimental results showing that l-lactate enters mitochondria under conditions where pyruvate is a non-penetrant compound (21) or where the pyruvate/H^+^ symporter is blocked by an inhibitor (6). These experimental approaches can be also applied to measurements of oxygen consumption (in the presence or absence of ADP), proton efflux and membrane potential generation in the future. By applying the control strength criterion with various non-penetrant inhibitors (19) it can be established whether or not the rate of the above processes mirrors that of l-lactate transport across the mitochondrial membrane. Thus, l-lactate transport can be investigated quantitatively, including the occurrence of hyperbolic kinetics, pH profile, etc. Moreover, comparison made between the inhibition profiles of pyruvate and l-lactate-dependent mitochondrial processes through the use of compounds that are unable to enter mitochondria allows for a distinction between l-lactate and pyruvate carriers.

Briefly, it has also been shown that externally added l-lactate can enter both normal and cancer prostate cells and in particular, in a carrier-mediated manner, enters their mitochondria, where an l-LDH exists and is located in the inner compartment. The m-l-LDHs have been demonstrated to differ from the cytosolic enzymes that themselves differ from one another. Normal and cancer cells show differences with respect to m-l-LDH protein level and activity, where both the enzyme expression and activity are higher in cancer cells.

In 2011, the existence of monocarboxylate transporter (MCT) and LDH proteins in mitochondrial reticula of breast cancer cell lines was demonstrated (22). In that case, the expression of both MCTs and l-LDH was measured, and their mitochondrial localization was determined *via* immunofluorescence, a technique that does not allow for the identification of the submitochondrial localization.

A broader investigation of l-lactate transport and metabolism in cancer cell mitochondria was carried out in human hepatocellular carcinoma (Hep G2) cells (21) in which gluconeogenesis takes place (23). Hep G2 cell mitochondria (Hep G2-M) possess an m-l-LDH restricted to the inner mitochondrial compartment. Cytosolic and mitochondrial l-LDHs were also found to differ from one another in their saturation kinetics. The occurrence of a carrier-mediated l-lactate transport in these mitochondria has also been shown. Importantly, the efflux of various metabolites, including pyruvate, oxaloacetate, malate, and citrate, resulting from l-lactate addition to mitochondria was first shown, this giving a first insight into the role of mitochondrial metabolism of l-lactate; accordingly, the occurrence of an l-lactate/pyruvate shuttle devoted to the oxidation of the cytosolic NADH was also shown. Ultimately, the removal of the oxidation product by carrier-mediated transport and mitochondrial metabolism overcomes any theoretical thermodynamic difficulty which was considered to rule out any l-lactate oxidation in the mitochondria.

These findings strongly suggest that a revision of the dogmatic view of glucose metabolism is needed with a special focus on the role of l-lactate and m-l-LDH in gluconeogenesis. Hence, the Cori cycle (formulated in 1929 as an energy-requiring metabolic pathway in animals, where carbon atoms of glucose pass along the circular route: muscle glycogen → blood lactate → liver (where gluconeogenesis occurs) → blood glucose → muscle glucose → muscle glycogen) demands revision, too. In this regard, cellular l-lactate oxidation, which is necessary for the production of glucose in the Cori cycle, has been traditionally postulated to take place in the cytosol, but is it? The cytosolic-l-LDH (c-l-LDH) is a reducing enzyme, the final step of the glycolytic pathway, which converts pyruvate to l-lactate, and thus provides the regeneration of NAD^+^. This reaction should proceed unabated, independently of the presence or absence of oxygen, as the standard free-energy $\left(\Delta G^{0^{\prime }}\right)$ change of pyruvate conversion to l-lactate is about −6 kcal/mol. In addition, the high affinity of pyruvate to c-l-LDH would explain the fact that the normal [l-lactate]/[pyruvate] ratio in blood and other tissues is >10, a value that cannot correspond with the proposal of pyruvate as the end product of glycolysis under normal conditions. Therefore, the dogmatic portrayal of this reaction as bidirectional is misleading and has been accepted to date due to the absence of a possible alternative. We contend that l-lactate oxidation back to pyruvate does not take place in the cytosol, but rather, it occurs in the mitochondria. Indeed, there are only two options to prevent l-lactate accumulation in the cytosol, either l-lactate is transported out of the cell (under anaerobic conditions) and/or is oxidized *via* m-l-LDH upon its transport into the mitochondrion (under aerobic conditions). Therefore, even if we agree with Lu et al. (24) that “the majority of glycolysis-derived pyruvate is diverted to lactate fermentation,” we cannot accept that l-lactate is “kept away from mitochondrial oxidative metabolism.”

**Figure 1:**
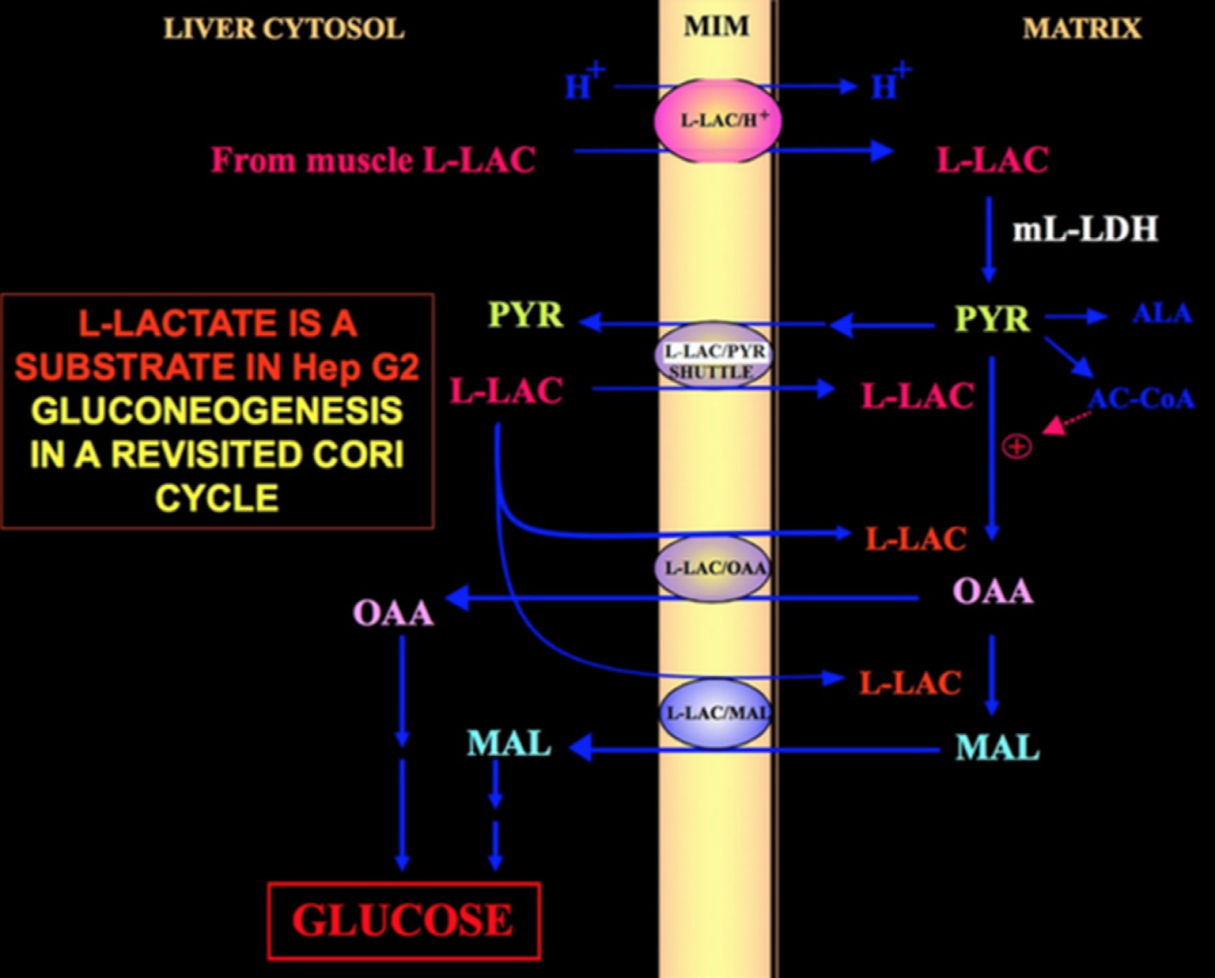
Cori cycle revisited in Hep G2 cells. Given that pyruvate cannot enter Hep G2-M, as shown in Pizzuto et al. (21), l-lactate produced in the muscles reaches the liver *via* the blood stream and from the cytosol enters mitochondria; in the matrix l-lactate metabolism gives rise to pyruvate (PYR) *via* m-L-LDH and then to oxaloacetate (OAA) and malate (MAL) that are exported from the mitochondria to the cytosol via three putative carriers to be used for the l-lactate pyruvate shuttle and for gluconeogenesis to occur via a mechanism similar to that already shown by de Bari et al. (6).

Of special interest is the fact that pyruvate cannot enter Hep G2-M. In fact, contrary to malate + glutamate and l-lactate, externally added pyruvate fails to cause either oxygen consumption or membrane potential generation [see Pizzuto et al. (21) for details]. Notice that an impairment of pyruvate transport in cancer cells has been reported by Paradies et al. (25). Therefore, independently of the theoretical unfeasibility of l-lactate oxidation in the cytosol, as was explained above, the classic Cori cycle cannot occur in Hep G2cells. Therefore, we offer a revised Cori cycle (Figure 1), which involves both the mitochondrial carriers that mediate the l-lactate-dependent traffic and the m-l-LDH, which provides pyruvate inside mitochondria. Accordingly, the appearance outside mitochondria of oxaloacetate and malate derived from l-lactate uptake and metabolism *via* m-l-LDH, pyruvate dehydrogenase, pyruvate carboxylase, and malate dehydrogenase and by exchanges, likely due to the l-lactate/oxaloacetate and l-lactate/malate antiporters, confirms an anaplerotic role for l-lactate in gluconeogenesis in which mitochondria play a unique role. Importantly, the addition of l-lactate to Hep G2-M results in the appearance outside mitochondria of citrate, the fatty acid precursor. Accordingly, by using high-resolution mass spectrometry, l-lactate uptake into mitochondria of HeLa and H460 cells was found and proved to result in lipid synthesis; additionally, transmission electron microscopy confirmed that LDH is localized to the mitochondria (26). Surprisingly, the anaplerotic role of l-lactate mitochondrial metabolism has not been considered when cancer metabolism was “reexamined” (27).

We believe that the proposed revision of the Cori cycle, necessary for Hep G2 cells, should also be considered in all other types of cells where mitochondrial metabolism of l-lactate is active. For instance, partial reconstruction of *in vitro* gluconeogenesis arising from mitochondrial l-lactate uptake/metabolism was shown in the absence of LDH outside mitochondria (6).

The role of the mitochondrial l-lactate metabolism merits further focus: given that hydrogen peroxide production in the tumor microenvironment fuels the anabolic growth of cancer cells (28), a possible role of the putative mitochondrial l-lactate oxidase (LOX) which generates hydrogen peroxide in rat liver mitochondria (29) should be investigated; the LOX existence in Hep G2-M appears to be consistent with the evidence that rotenone, which blocks oxygen consumption induced by the addition of malate + glutamate fails to inhibit oxygen consumption induced by the addition of l-lactate.

## Author Contributions

SP conceived this opinion, shared it and wrote the paper with AS.

## Conflict of Interest Statement

The authors declare that the research was conducted in the absence of any commercial or financial relationships that could be construed as a potential conflict of interest. The reviewer [GP] declared a shared affiliation, with no collaboration, with one of the authors [SP] to the handling Editor.
